# Supplementary material for: Forces during cellular uptake of viruses and nanoparticles at the ventral side
Source: Nat Commun. 2020 Jan 2;11:32. doi: 10.1038/s41467-019-13877-w (PMC6940367; doi:10.1038/s41467-019-13877-w)
Supplement: Supplementary file 1 — Supplementary Information [file 41467_2019_13877_MOESM1_ESM.pdf]

Supplementary information

Forces during cellular uptake of viruses and nanoparticles at the ventral side

Wiegand et al.

This document contains:

- 7 supplementary figures
- 1 supplementary table
- 2 supplementary notes
- supplementary references

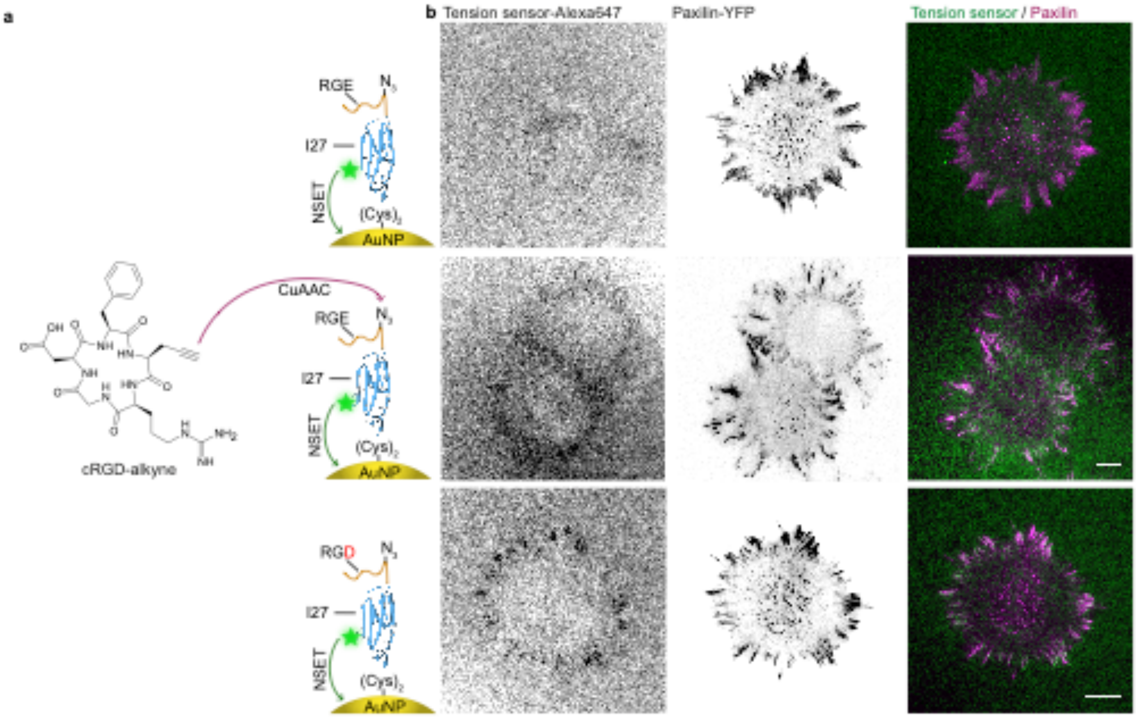

Supplementary Figure 1: Randomly labeled titin-based tension probes bearing RGD ligands report integrin mediated forces.

a, Schematic of the I27 tension sensors expressing the peptide sequence RGE at the N-terminus as negative control (upper row), RGE with clicked cRGD-alkyne (middle row) and RGD (lower row), respectively. Tension sensors were labeled with Alexa647-NHS, incubated on glass substrates with randomly immobilized gold nanoparticles (AuNPs) and optionally cRGD-alkyne was clicked to the *p*-azidophenylalanine (middle row). Rat embryonic fibroblasts (REF) were seeded on the substrates 1 h prior to imaging to compare with previous results[1]. **b,** Representative fluorescence widefield images of fluorescently labeled tension sensors (green) and REF cells, expressing paxilin-YFP (magenta), adhering to substrates (inverted LUT in separate images). Scale bars = 10 µm.

REFs seeded on Alexa647-I27-RGE surfaces (upper row) were able to spread on surfaces and formed focal adhesions but did not induce force signals of the tension probes. On both, Alexa647-I27-RGD and Alexa647-I27-RGE + clicked cRGD-alkyne tension sensors (middle and lower row), we observed a typical spatial distribution of integrin-mediated forces at focal adhesion sites. This validates that the tension probes with clicked ligands detect specific forces with values exerted by individual integrin receptors exceeding ~ 40 pN, which is in agreement with the forces reported for single integrins by other techniques[2–4].

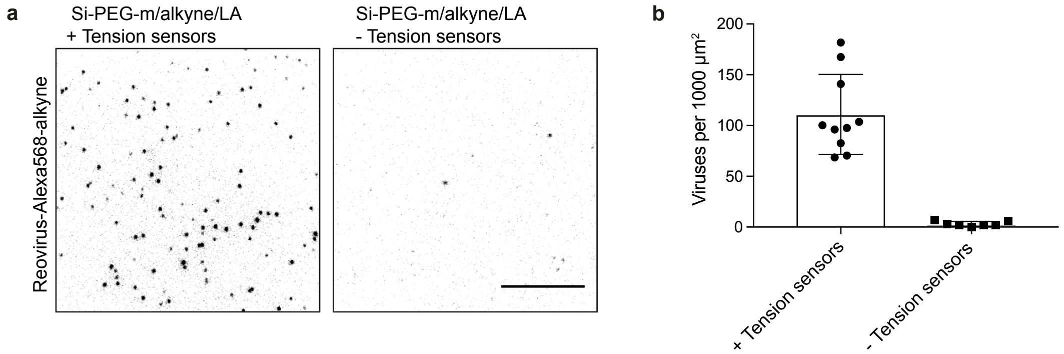

Supplementary Figure 2: Alkyne-modified reovirus are specifically immobilized on molecular force sensors via click chemistry.

a, Representative TIRF images of reoviruses labeled with Alexa568 and alkyne linkers clicked on SiO_2-PEG-methoxy/lipoic acid/alkyne surfaces with tension sensors (AuNPs + Alexa647-I27-RGE) or without (inverted LUT). Scale bar = 10 µm. **b**, Quantification of surface-bound virus particles from fluorescent images per 1000 µm^2. Data are shown as mean value ± s.d., *n* = 10 ROI from three technical repeats. Source data are provided as a source data file.

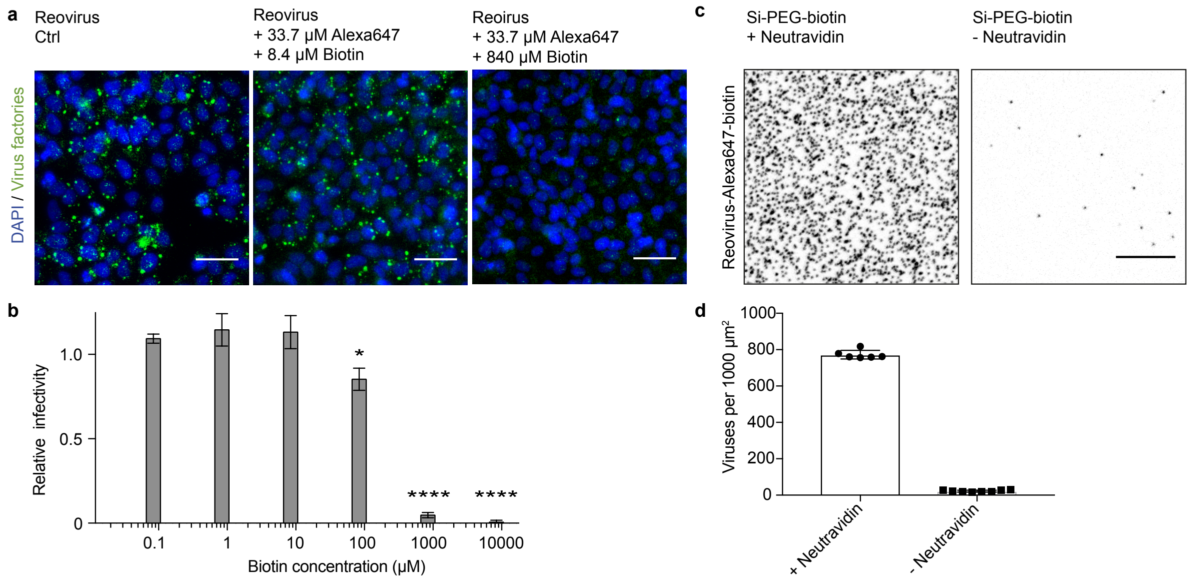

Supplementary Figure 3: Biotinylated reoviruses are infectious and can specifically be immobilized via biotin-neutravidin.

a, Reovirus particles were labeled with Alexa647-NHS at $c_{final,Alexa647}$ = 33.7 µM and biotin-NHS between 0 and 8.4 mM. HeLa cells infected with the indicated virus solutions were immune stained for virus factories (green) and for cell nuclei with DAPI (blue) and imaged with widefield fluorescence microscopy. Scale bar = 50 µm. **b**, Percentage of infected cells was normalized to the percentage of cells infected with non-modified virus. *, $P < 0.0376$ and ****, $P < 0.0001$, Students *t*-test in comparison to non-modified virus infected cells. Data are shown as mean value ± s.d., $n = 6$ ROI from two technical repeats. **c**, Inverted fluorescence confocal images of biotinylated viruses ($c_{final,biotin}$ = 8.4 µM) on Silane-PEG-biotin coated surfaces incubated with or without neutravidin. Scale bar = 10 µm. **b**, Quantification of surface-bound virus particles from fluorescent images per 1000 µm^2. Data are shown as mean value ± s.d., $n = 10$ ROI from two technical repeats. Source data are provided as a source data file.

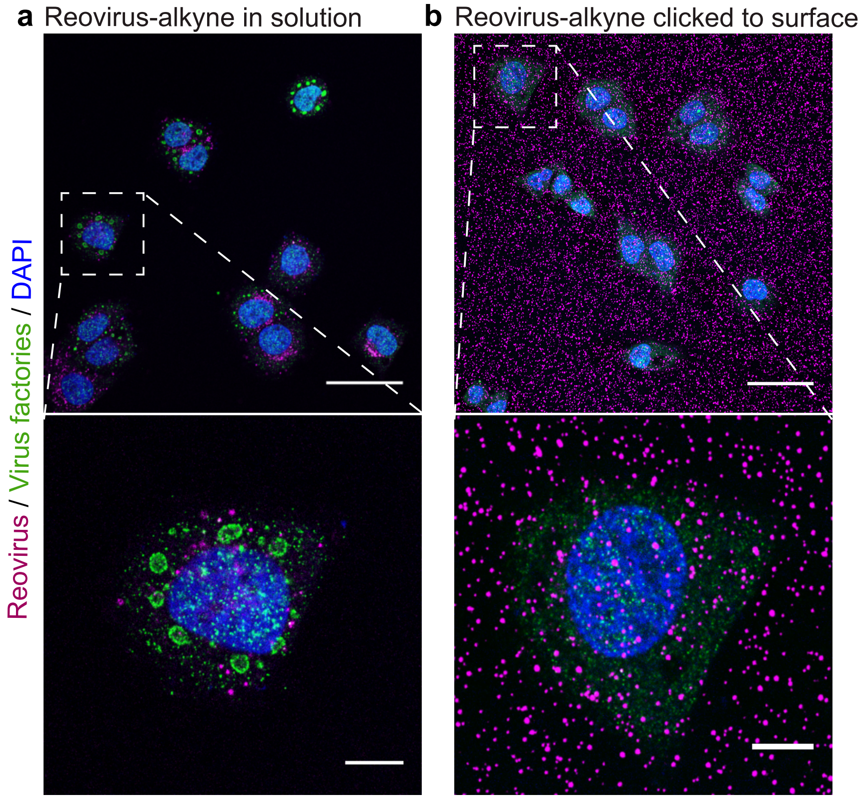

Supplementary Figure 4: Covalently bound reoviruses cannot be removed from the surface.

a, Alkyne-modified reovirus particles (Alexa568, magenta) were applied in solution or **b**, covalently immobilized via click chemistry as previously reported[5]. HeLa cells seeded on the surfaces overnight were fixed and stained for virus factories (green) and DAPI (blue). Scale bars = 50 µm and 10 µm in the zoomed images.

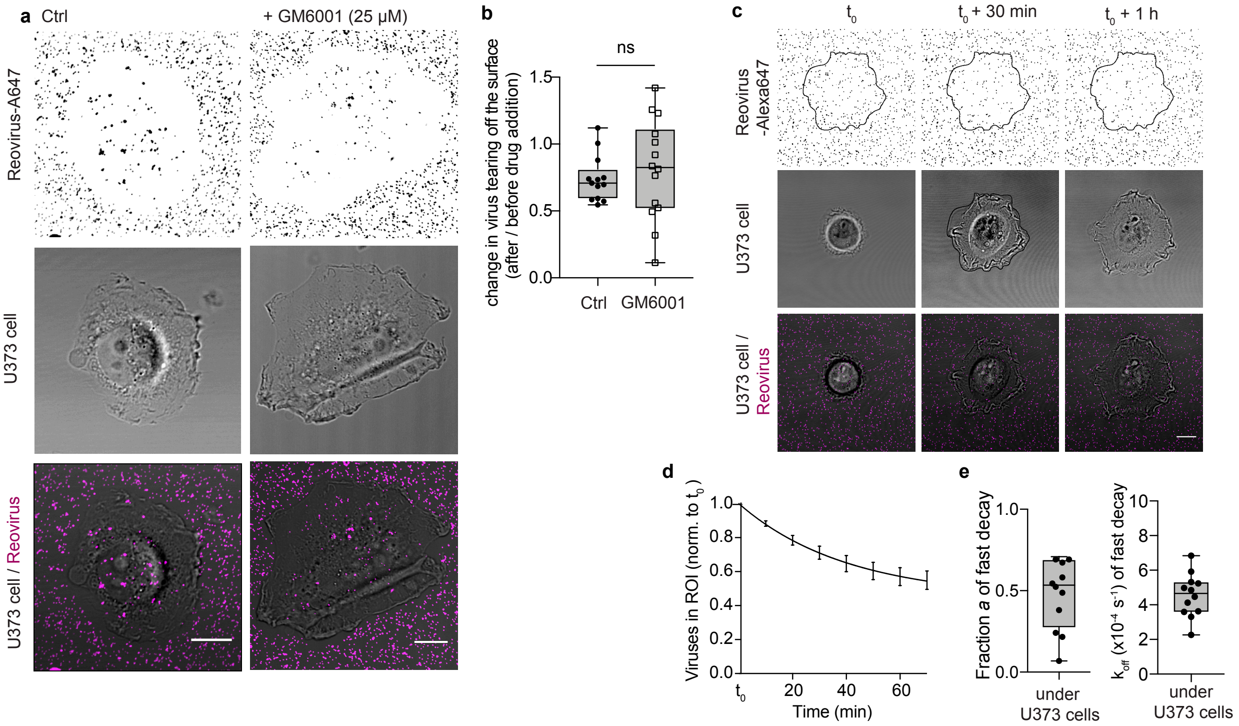

Supplementary Figure 5: Biotin-neutravidin immobilized reoviruses are torn off protease independently by U373 cells.

a, Biotin-modified reovirus particles (Alexa647, magenta) were non-covalently immobilized via neutravidin on a biotinylated PEG layer. U373 cells (transmission, grey) were seeded on the surface and after 30 min the maxtrix metalloproteinase inhibitor GM6001 was added optionally at 25 µM final concentration. Cells were fixed after 4 h and observed by confocal microscopy. Scale bars, 10 µm. **b**, Change in virus tearing off the surface by U373 cells was evaluated by the relative number of viruses being removed during the 30 min after the addition of GM6001 divided by the relative number during the 30 min before drug addition. (*n* = 13 and 14 cells, respectively, 2 technical repeats, ns = not significant according to unpaired two-tailed Mann-Whitney test) **c**, Confocal time-lapse images of a U373 cell spreading and tearing off reovirus particles from the surface with cell outline 1 h post seeding defining the region of interest (ROI). Scale bar, 10 µm. **d**, Normalized number of virus particles in ROI under U373 cells over time. (*n* = 13 cells, 2 technical repeats, mean ± s.e.m, two-phase exponential decay fit). **e**, Fitting parameters *a*, the fraction, and k_{off} the off rate of particles being actively removed by HeLa cells from the two-phase-decay of particles in the ROI. (Box-plots represent median ± 95 % CI with whiskers to the min and max values.) Source data are provided as a source data file.

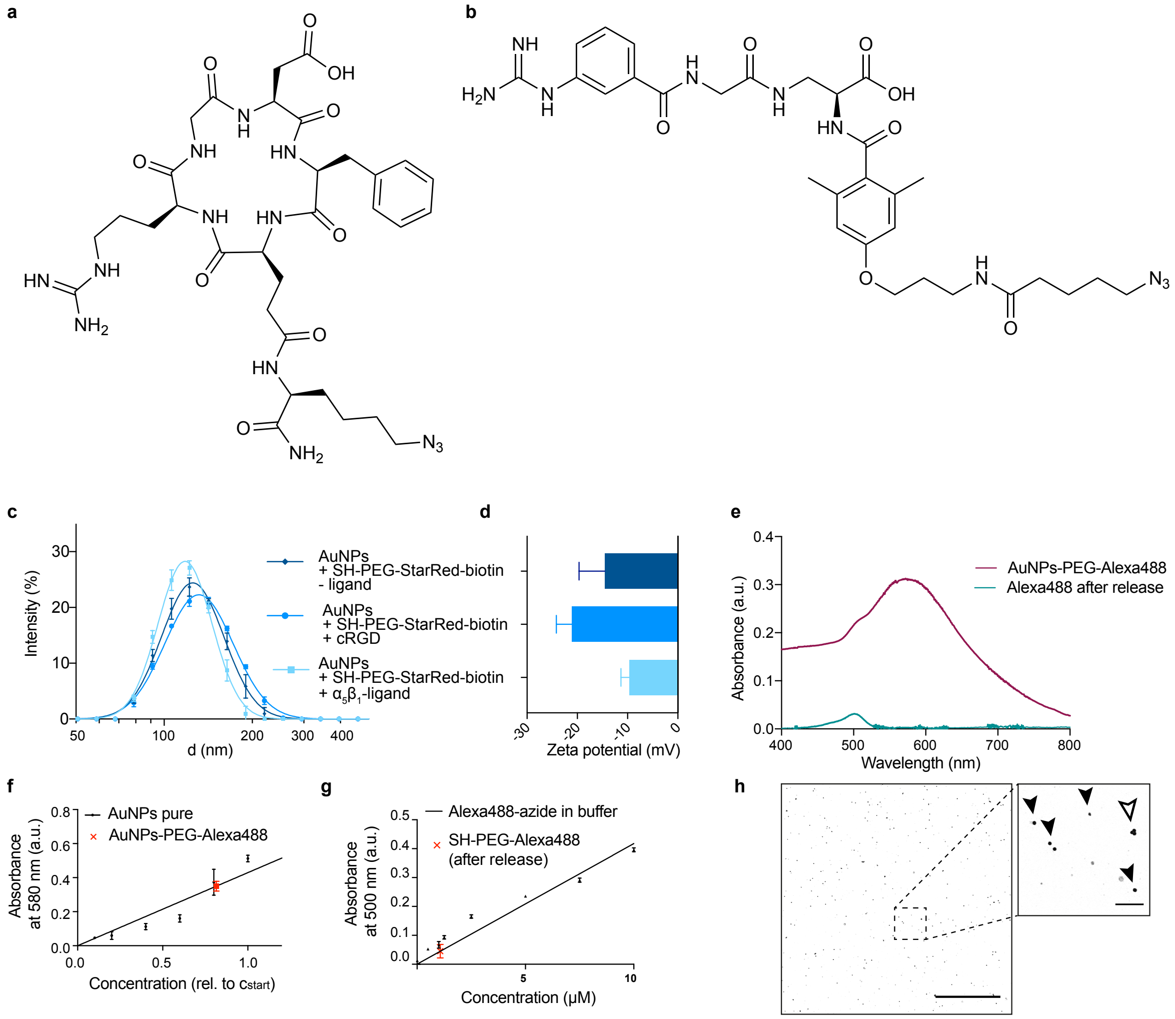

Supplementary Figure 6: Characterization of modified gold nanoparticles.

AuNPs were modified with SH-PEG partially labeled with the dye StarRed or alkyne functionalized (1:10). Biotin-azide and optionally **a,** cRGD-azide, **b**, an integrin $\alpha_5\beta_1$-selective ligand[6] or Alexa488-azide was covalently bound via CuAAC. **c**, For dynamic light scattering and zeta-potential measurements particles were diluted 1:10 in PBS. Log-normal distribution of particle diameter revealed a mode of (127 ± 3) nm for all AuNPs. **d,** Zeta-potentials for AuNPs were -15 (± 5) mV, -21 (± 3) mV and -10 (± 1) mV for the particles with SH-PEG-StarRed-biotin without ligands and additionally +cRGD or +beta1 ligands, respectively. n = 3 independent measurements. **e,** UV-Vis spectroscopy of AuNPs and Alexa488-azide after release from the particles by 0.1 M DTT[7]. Calibration curve of **f**, AuNPs and **g**, Alexa488-N3 in click buffer. 1.07 µM Alexa488-azide was detected per $3.096 \cdot 10^9$ AuNPs per ml resulting in a final number of $\sim 2 \cdot 10^5$ ligands per AuNP. Data are represented as mean ± s.d. **h**, SEM images (inverted LUT) of biotin-neutravidin immobilized AuNPs on PEGylated glass surfaces. Arrowheads point towards single AuNPs, hollow arrow head points to a 3-particle aggregate. Scale bars are 10 µm in the overview and 1 µm in the zoom-in. Source data are provided as a source data file.

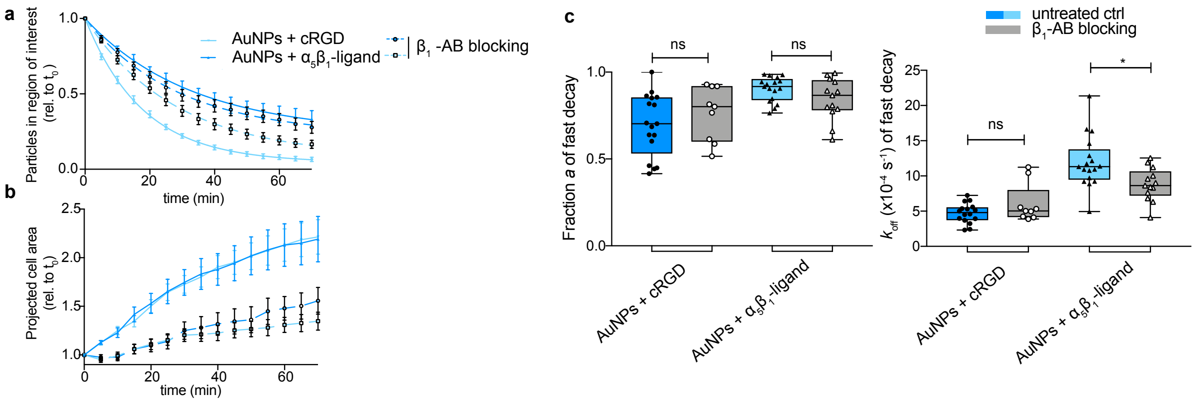

Supplementary Figure 7: Tearing off nanoparticles with specific receptors for interaction with HeLa cells.

a, Normalized number of nanoparticles in ROI under HeLa cells with or without antibody (AB) blocking of integrin β_1 over time. AB blocking of integrin β_1 HeLa cells reduced uptake of nanoparticles coated with integrin $\alpha_5\beta_1$-selective ligands (light blue), while uptake of nanoparticles with cRGD was unchanged. Data are represented as mean ± s.e.m. **b**, AB-blocking of integrin β_1 reduces the overall cell spreading. **c,** Blocking of integrin β_1 did not change the uptake rate for AuNPs with cRGD but it reduced significantly the uptake kinetics of AuNPs with integrin $\alpha_5\beta_1$-selective ligands. (n = 16, 9, 17, 13 cells, 3 technical repeats, box-plots represent median ± 95 % CI with whiskers to the min and max values, one-way ANOVA with post-hoc Tukey test, *, P = 0.0225). Source data are provided as a source data file.

Supplementary Table 1: Modelling parameters

Parameter	Value	Reference
Nanoparticle hydrodynamic radius R_{np}	64 nm	Measured by DLS (Fig. S6)
Reovirus hydrodynamic radius R_{rv}	45 nm	Measured by DLS in [5]
Bending rigidity κ	25 k_BT	[8,9]
Surface tension σ	10^{-5} N m^{-1}	[10]
Linear dimension of neutravidin d_{na}	5 - 8 nm	[11]
Distance between cell membrane and ECM	80 nm	[12]
Rupture length z	10 nm	estimated

Supplementary Note 1

Ligand density on AuNPs was determined to be ~$2\cdot10^5$ per AuNP by UV-Vis spectroscopy of an organic dye (Alexa488)-azide, which was coupled to the PEG-alkyne modified AuNPs and subsequently released by DTT treatment (Supplementary Fig. 7). For AuNPs with a diameter of 127.3 nm (A = $5.1\cdot10^{-14}$ m^2) this equals ~$4\cdot10^{18}$ ligands per AuNPs. Further the ligand density can be estimated from previous characterizations on self-assembled PEG-alkyne layers in 2D by Schenk *et al.*[13]. For a homogeneous PEG-alkyne layer a peptide density of $2.4\cdot10^{17}$ ligands per m^2 was quantified by fluorescence spectroscopy in a chymotrypsin digestion assay. The discrepancy in the values could arise from a higher binding rate of the small dye molecules compared to cRGD ligands, higher binding efficiency on the particles compared to a 2D substrate or from impurities of free Alexa488-azide, which remained in solution after purification of the AuNPs by size exclusion columns.

However, the effective ligand density is limited by the number of cellular receptors available in the plasma membrane. The dimension of integrins in the cell membrane itself is between 6 – 15 nm[14,15] depending on the activation state. For a dense packing of integrins with minimum distance between two integrin heads of 20 nm[16], this would equal a maximal density of $(2/\sqrt{3})/(10^2\ \mathrm{nm}^2) \approx 10^{16}$ receptors per m^2, out of which only a fraction is actively binding ligands. But, a high ligand density on the AuNPs could still improve availability of ligands and thus increase the adhesion strength between the particles and the cells.

Supplementary Note 2

Reovirus capsid has in total 12 dominant ligands σ1 located at the each of the 12 pentagonal faces that make up the virus capsid. They are known to interact with the cellular receptor JAM-A with a dissociation constant $K_d = 9\cdot10^{-8}$ M.[17] This gives a binding energy of roughly

$\Delta\epsilon_{\sigma1\text{-JAM-A}} = k_BT \ln(K_d/c_0) \approx 16\ k_BT$ per receptor, with c_0 = 1 M. Additionally, the outer capsid protein λ_2 displays the integrin-binding sequence RGD leading to another potential binding of maximal 5 $ß_1$-integrins per face[18,19], which gives a total of 72 specific ligands on

the virus capsid. The binding energy of this receptor-ligand pair has not been measured yet. We assume it has the same binding energy as the σ1-JAM-A interaction, which is further corroborated by the structural similarities with adenovirus. Interactions between $\alpha_v ß_3$ integrins and a RGD motive in the adenovirus penton base have been quantified with $\Delta\epsilon_{\text{integrin-adenovirus}} \approx 15\ k_BT$.[20] Hence, the specific adhesion density we found from biotin-neutravidin immobilized virus uptake corresponds to $N = (\Delta W/\Delta\epsilon)\cdot 4\pi R^2 =$ $(0.08\ \text{mJ/m}^2\ /\ 16\ k_BT) \cdot 4\pi\ 45^2\ \text{nm} = 30$ receptor ligand interactions. However, *in vivo* steric hindrance and availability of ligands will limit this number, which could be compensated by stronger non-specific adhesions e.g. between viral capsid proteins sialic acids.

Supplementary references

1. Galior, K., Liu, Y., Yehl, K., Vivek, S. & Salaita, K. Titin-Based Nanoparticle Tension Sensors Map High-Magnitude Integrin Forces within Focal Adhesions. *Nano Lett.* **16**, 341–348 (2016).
2. Wang, X. & Ha, T. Defining Single Molecular Forces Required to Activate Integrin and Notch Signaling. *Science (80-.).* **7219**, 991–994 (2013).
3. Morimatsu, M., Mekhdjian, A. H., Adhikari, A. S. & Dunn, A. R. Molecular tension sensors report forces generated by single integrin molecules in living cells. *Nano Lett.* **13**, 3985–9 (2013).
4. Jurchenko, C., Chang, Y., Narui, Y., Zhang, Y. & Salaita, K. Integrin-generated forces lead to streptavidin-biotin unbinding in cellular adhesions. *Biophys. J.* **106**, 1436–46 (2014).
5. Fratini, M. *et al.* Surface Immobilization of Viruses and Nanoparticles Elucidates Early Events in Clathrin-Mediated Endocytosis. *ACS Infect. Dis.* **4**, 1585–1600 (2018).
6. Heckmann, D. *et al.* Rational Design of Highly Active and Selective Ligands for the α5β1 Integrin Receptor. *ChemBioChem* **9**, 1397–1407 (2008).
7. Thaxton, C. S., Hill, H. D., Georganopoulou, D. G., Stoeva, S. I. & Mirkin, C. A. A Bio-Bar-Code Assay Based upon Dithiothreitol-Induced Oligonucleotide Release. *Anal. Chem.* **77**, 8174–8178 (2005).
8. Boal, D. *Mechanics of the cell, second edition. Mechanics of the Cell, Second Edition* **571**, (2012).
9. Kumar, G. & Sain, A. Shape transitions during clathrin-induced endocytosis. *Phys. Rev. E* **94**, (2016).
10. Saleem, M. *et al.* A balance between membrane elasticity and polymerization energy sets the shape of spherical clathrin coats. *Nat. Commun.* **6**, 6249 (2015).
11. Pazy, Y. *et al.* Dimer-tetramer transition between solution and crystalline states of streptavidin and avidin mutants. *J. Bacteriol.* **185**, 4050–4056 (2003).
12. Dejardin, M.-J. *et al.* Lamellipod Reconstruction by Three-Dimensional Reflection Interference Contrast Nanoscopy (3D-RICN). *Nano Lett.* **18**, 6544–6550 (2018).
13. Schenk, F. C., Boehm, H., Spatz, J. P. & Wegner, S. V. Dual-functionalized nanostructured biointerfaces by click chemistry. *Langmuir* **30**, 6897–905 (2014).
14. Xiong, Hi.-P. *et al.* Crystal Structure of the Extracellular Segment of Integrin alpha V beta 3. *Science (80-.).* **294**, 339–345 (2001).
15. Hynes, R. O. Integrins: Versatility, modulation, and signaling in cell adhesion. *Cell* **69**, 11–25 (1992).
16. Erb, E. M., Tangemann, K., Bohrmann, B., Müller, B. & Engel, J. Integrin αIIβ3 reconstituted into lipid bilayers is nonclustered in its activated state but clusters after fibrinogen binding. *Biochemistry* **36**, 7395–7402 (1997).
17. Barton, E. S. *et al.* Junction adhesion molecule is a receptor for reovirus. *Cell* **104**, 441–451 (2001).
18. Seliger, L. S., Zheng, K. & Shatkin, A. J. Complete nucleotide sequence of reovirus L2 gene and deduced amino acid sequence of viral mRNA guanylyltransferase. *J. Biol. Chem.* **262**, 16289–93 (1987).
19. Maginnis, M. S. *et al.* Beta1 Integrin Mediates Internalization of Mammalian Reovirus. *J. Virol.* **80**, 2760–70 (2006).
20. Veesler, D. *et al.* Single-particle EM reveals plasticity of interactions between the adenovirus penton base and integrin V 3. *Proc. Natl. Acad. Sci.* **111**, 8815–8819 (2014).
