# Supplementary material for: Forces during cellular uptake of viruses and nanoparticles at the ventral side
Source: Nat Commun. 2020 Jan 2;11:32. doi: 10.1038/s41467-019-13877-w (PMC6940367; doi:10.1038/s41467-019-13877-w)
Supplement: Supplementary file 13 — Reporting Summary [file 41467_2019_13877_MOESM13_ESM.pdf]

nature research

Corresponding author(s): Tina Wiegand

Last updated by author(s): Nov 23, 2019

Reporting Summary

Nature Research wishes to improve the reproducibility of the work that we publish. This form provides structure for consistency and transparency in reporting. For further information on Nature Research policies, see Authors & Referees and the Editorial Policy Checklist .

Statistics

For all statistical analyses, confirm that the following items are present in the figure legend, table legend, main text, or Methods section.

n/a	Confirmed	
☐	☒	The exact sample size (n) for each experimental group/condition, given as a discrete number and unit of measurement
☐	☒	A statement on whether measurements were taken from distinct samples or whether the same sample was measured repeatedly
☐	☒	The statistical test(s) used AND whether they are one- or two-sided<br>*Only common tests should be described solely by name; describe more complex techniques in the Methods section.*
☒	☐	A description of all covariates tested
☐	☒	A description of any assumptions or corrections, such as tests of normality and adjustment for multiple comparisons
☐	☒	A full description of the statistical parameters including central tendency (e.g. means) or other basic estimates (e.g. regression coefficient) AND variation (e.g. standard deviation) or associated estimates of uncertainty (e.g. confidence intervals)
☐	☒	For null hypothesis testing, the test statistic (e.g. F, t, r) with confidence intervals, effect sizes, degrees of freedom and P value noted<br>*Give P values as exact values whenever suitable.*
☒	☐	For Bayesian analysis, information on the choice of priors and Markov chain Monte Carlo settings
☒	☐	For hierarchical and complex designs, identification of the appropriate level for tests and full reporting of outcomes
☒	☐	Estimates of effect sizes (e.g. Cohen's d, Pearson's r), indicating how they were calculated

Our web collection on statistics for biologists contains articles on many of the points above.

Software and code

Policy information about availability of computer code

Data collection	No software besides microscope control was employed for data collection.
Data analysis	Custom code in Matlab (MathWorks) was employed to align spectral channels by projective transformation and to generate fluorescent tracks of molecular tension probes and virus particles and is deposited with the identifiers: 10.5281/zenodo.3551377, 10.5281/zenodo.3556565<br>Ilastik 1.3.0 (open source) was employed for single particle tracking.<br>Custom code in Python was employed to analyze the particle tracks and is deposited with the identifier 10.5281/zenodo.3551377.<br>Prism 7 (Graphpad Software) was employed for statistical analysis and fitting of data.

For manuscripts utilizing custom algorithms or software that are central to the research but not yet described in published literature, software must be made available to editors/reviewers. We strongly encourage code deposition in a community repository (e.g. GitHub). See the Nature Research guidelines for submitting code & software for further information.

Data

Policy information about availability of data

All manuscripts must include a data availability statement. This statement should provide the following information, where applicable:

- Accession codes, unique identifiers, or web links for publicly available datasets
- A list of figures that have associated raw data
- A description of any restrictions on data availability

The data that support the findings of this study are available from the corresponding authors upon request.

Field-specific reporting

Please select the one below that is the best fit for your research. If you are not sure, read the appropriate sections before making your selection.

☒ Life sciences ☐ Behavioural & social sciences ☐ Ecological, evolutionary & environmental sciences

For a reference copy of the document with all sections, see nature.com/documents/nr-reporting-summary-flat.pdf

Life sciences study design

All studies must disclose on these points even when the disclosure is negative.

Sample size	No statistical methods were used to predetermine sample size. During experiments, we determined that a sample size of about 10-30 cells with multiple single (virus) particles binding (as indicated) was sufficient to assess whether the differences between conditions were statistically significant.
Data exclusions	No data were excluded from the analysis but for presentation of variability between cells, the fitting parameters "koff" and "a" were plotted from individual fits per cell in Figs. 2e, 3d, 4e, 5d, S5 and S7 excluding these fits that were either hit-constraint or ambiguous.
Replication	All experimental findings were reproduced independently at least two times.
Randomization	Cells were randomly selected per condition.
Blinding	Investigators were not blinded for different experimental conditions. Investigators were blinded for analysis of fluorescent tracks in background vs. underneath cells.

Reporting for specific materials, systems and methods

We require information from authors about some types of materials, experimental systems and methods used in many studies. Here, indicate whether each material, system or method listed is relevant to your study. If you are not sure if a list item applies to your research, read the appropriate section before selecting a response.

Materials & experimental systems

n/a	Involved in the study
☐	☒ Antibodies
☐	☒ Eukaryotic cell lines
☒	☐ Palaeontology
☒	☐ Animals and other organisms
☒	☐ Human research participants
☒	☐ Clinical data

Methods

n/a	Involved in the study
☒	☐ ChIP-seq
☒	☐ Flow cytometry
☒	☐ MRI-based neuroimaging

Antibodies

Antibodies used	anti µNS rabbit IgG (generated by GenScript), human integrin beta1 (CD29) blocking antibody clone P5D2 (#MAB17781, R&D systems, USA)
Validation	All antibodies are validated for the species and assay used as described in the manufacturer's web page.

Eukaryotic cell lines

Policy information about cell lines

Cell line source(s)	BSC1 stably expressing σ2-eGFP were described previously (Ref 60 in the paper). HeLa cells were purchased from ATCC. Rat embryonic fibroblasts (REF) stably expressing paxilin-YFP were originally obtained from Benny Geiger (Weizman Institute, Israel). U373-MG were originally obtained from Tomas Kirchhausen (Harvard Medical School, Boston, USA).
Authentication	Cell lines were not authenticated.
Mycoplasma contamination	All cell lines tested negative for mycoplasma contamination.
Commonly misidentified lines (See ICLAC register)	U-373 MG cells were used as control for reovirus uptake as previously employed in Ref 21 in the paper.
