# Supplementary material for: Forces during cellular uptake of viruses and nanoparticles at the ventral side
Source: Nat Commun. 2020 Jan 2;11:32. doi: 10.1038/s41467-019-13877-w (PMC6940367; doi:10.1038/s41467-019-13877-w)
Supplement: Supplementary file 14 — Description of Additional Supplementary Files [file 41467_2019_13877_MOESM14_ESM.pdf]

Title: Movie 1: Titin-based molecular tension sensors detects forces on single virus exerted by the ventral cell side.
Description: TIRF movie of tension sensors (Alexa647-I27-RGE), virus particles (reovirus-Alexa568) and AP2-GFP signal of a BSC1 cell spreading on the tension sensor surface (grouped z-project à 3 slides, all channels with inverted LUT). Scale bar, 10 µm.

Title: Movie 2: Tearing off biotin-neutravidin immobilized virus particles by U373 cells is independent of matrix metalloproteinases.
Description: Confocal time-lapse movie of U373 cells (transmission, left) spreading on a surface with biotin-neutravidin immobilized reovirus particles (Alexa647, right, inverted LUT). After 30 min the matrix metalloproteinase inhibitor GM6001 was added at 25 µM. Scale bar, 10 µm.

Title: Movie 3: Tearing off biotin-neutravidin immobilized virus particles by HeLa cells.
Description: Confocal time-lapse of HeLa cells (transmission) tearing off biotin-neutravidin immobilized reovirus particles (Alexa647, inverted LUT). Scale bar, 10 µm.

Title: Movie 4: Tearing off biotin-neutravidin immobilized particles by HeLa cells shows spatial preference underneath cell periphery.
Description: Interference reflection microscopy time-lapse of HeLa cells (dark interference) tearing off biotin-neutravidin immobilized AuNPs (bright due to scattering). Scale bar, 10 µm.

Title: Movie 5: Tearing off biotin-neutravidin immobilized AuNPs by HeLa cells.
Description: Confocal time-lapse of HeLa cells (transmission) tearing off biotin-neutravidin immobilized AuNPs (StarRed, inverted LUT). Scale bar, 10 µm.

Title: Movie 6: Tearing off biotin-neutravidin immobilized AuNPs with cRGD ligands by HeLa cells.
Description: Confocal time-lapse of HeLa cells (transmission) tearing off biotin-neutravidin immobilized AuNPs with cRGD ligands (StarRed, inverted LUT). Scale bar, 10 µm.

Title: Movie 7: Tearing off biotin-neutravidin immobilized AuNPs with $\alpha_5\beta_1$-selective ligands by HeLa cells.
Description: Confocal time-lapse of HeLa cells (transmission) tearing off biotin-neutravidin immobilized AuNPs with $\alpha_5\beta_1$-selective ligands (StarRed, inverted LUT). Scale bar, 10 µm.

Title: Movie 8: Tearing off biotin-neutravidin immobilized AuNPs by HeLa cells with inhibition of actin polymerization by cytochalasin D.
Description: Confocal time-lapse of HeLa cells (transmission) tearing off biotin-neutravidin immobilized AuNPs (StarRed, inverted LUT). 1 h after seeding the cells actin polymerization inhibitor cytochalsin D was added at 60 nM final concentration. Scale bar, 10 µm.

Title: Movie 9: Tearing off biotin-neutravidin immobilized AuNPs by HeLa cells with inhibition of actin polymerization by latrunculin A.
Description: Confocal time-lapse of HeLa cells (transmission) tearing off biotin-neutravidin immobilized AuNPs (StarRed, inverted LUT). 1 h after seeding the cells actin polymerization inhibitor latrunculin A was added at 100 nM final concentration. Scale bar, 10 µm.

Title: Movie 10: Tearing off biotin-neutravidin immobilized AuNPs by HeLa cells with inhibition of ROCK by Y-27632.
Description: Confocal time-lapse of HeLa cells (transmission) tearing off biotin-neutravidin immobilized AuNPs (StarRed, inverted LUT). 1 h after seeding the cells ROCK inhibitor Y-27632 was added at 20 µM final concentration. Scale bar, 10 µm.
